# MIA is a potential biomarker for tumour load in neurofibromatosis type 1

**DOI:** 10.1186/1741-7015-9-82

**Published:** 2011-07-04

**Authors:** Mateusz Kolanczyk, Victor Mautner, Nadine Kossler, Rosa Nguyen, Jirko Kühnisch, Tomasz Zemojtel, Aleksander Jamsheer, Eike Wegener, Boris Thurisch, Sigrid Tinschert, Nikola Holtkamp, Su-Jin Park, Patricia Birch, David Kendler, Anja Harder, Stefan Mundlos, Lan Kluwe

**Affiliations:** 1 Institute of Medical Genetics, Charité, Universitätsmedizin Berlin, Humboldt University, Augustenburger Platz 1, D-13353 Berlin, Germany; 2 Development and Disease Group, Max Planck Institute for Molecular Genetics, Ihnestrasse 63-73, D-14195 Berlin, Germany; 3 Department of Oral and Maxillofacial Surgery, Department of Neurology, University Hospital Hamburg-Eppendorf, Martinistrasse 52, D-20246 Hamburg, Germany; 4 Department of Computational Molecular Biology, Max Planck Institute for Molecular Genetics, Ihnestrasse 63-73, D-14195 Berlin, Germany; 5 Center for Medical Genetics in Poznań, ul. Grudzieniec 4, 60-601 Poznań, Poland; 6 Department of Medical Genetics, Medical University of Poznan, 60-352 Poznań, Poland; 7 Institut für Klinische Genetik, Medizinische Fakultät Carl Gustav Carus, Technische Universität Dresden, Fetscherstrasse 74, 01307 Dresden, Germany; 8 Institute of Neuropathology, Charité-Universitätsmedizin Berlin, CVK, Augustenburger Platz 1, D-13353 Berlin, Germany; 9 Berlin Center for Regenerative Therapies, Charité-Universitätsmedizin Berlin Augustenburger Platz 1, D-13353 Berlin, Germany; 10 Department of Medical Genetics, University of British Columbia, Box 153, 4500 Oak Street, Vancouver, BC V6H 3N1, Canada; 11 Faculty of Medicine, University of British Columbia 600 - 1285 West Broadway Vancouver, BC V6H 3X8, Canada; 12 Institute of Neuropathology, University Hospital Münster, Domagkstrasse 19, D-48149 Münster, Germany; 13 German Cancer Research Center, Im Neuenheimer Feld, D-69120 Heidelberg, Germany

## Abstract

**Background:**

Neurofibromatosis type 1 (NF1) is a frequent genetic disease characterized by multiple benign tumours with increased risk for malignancy. There is currently no biomarker for tumour load in NF1 patients.

**Methods:**

*In situ *hybridization and quantitative real-time polymerase reaction were applied to investigate expression of cartilage-specific genes in mice bearing conditional inactivation of NF1 in the developing limbs. These mice do not develop tumours but recapitulate aspects of NF1 bone dysplasia, including deregulation of cartilage differentiation. It has been recently shown that NF1 tumours require for their growth the master regulator of cartilage differentiation SOX9. We thus hypothesized that some of the cartilage-specific genes deregulated in an Nf1Prx1 mouse model might prove to be relevant biomarkers of NF1 tumours. We tested this hypothesis by analyzing expression of the SOX9 target gene product melanoma-inhibitory activity/cd-rap (MIA) in tumour and serum samples of NF1 patients.

**Results:**

Increased expression of *Mia *was found in *Nf1*-deficient cartilage in mice. In humans, MIA was expressed in all NF1-related tumours and its serum levels were significantly higher in NF1 patients than in healthy controls. Among NF1 patients, MIA serum levels were significantly higher in those with plexiform neurofibromas and in those with large number of cutaneous (> 1,000) or subcutaneous (> 100) neurofibromas than in patients without such tumours. Most notably, MIA serum levels correlated significantly with internal tumour burden.

**Conclusions:**

MIA is a potential serum biomarker of tumour load in NF1 patients which could be useful in following the disease course and monitoring the efficacy of therapies.

## Background

Neurofibromatosis type 1 (NF1) is a genetic disorder resulting from mutations in the *NF1 *tumour suppressor gene. Susceptibility to neoplastic transformation is the main feature of the disease [1]. The most frequent tumours in NF1 are dermal neurofibromas, which can be found in more than 90% of adult patients [2]. Approximately 50% of NF1 patients develop plexiform neurofibromas (pNFs), which can undergo malignant transformation into malignant peripheral nerve sheath tumours (MPNSTs) [3-6]. MPNSTs are highly malignant tumours with a poor prognosis. The lifetime risk of developing MPNSTs in the NF1 patient is 8% to 13% [7].

Major challenges in clinical practice are to determine tumour burden and to monitor the disease course. While cutaneous neurofibromas are visible on physical examination, the diagnosis of pNFs, especially internal ones, depends on magnetic resonance imaging (MRI), which is costly and laborious. Furthermore, early diagnosis is crucial for complete resection of MPNSTs, which is up to now the only curative treatment [8]. A biomarker for assessment of tumour burden and detection of malignant transformation would therefore be of interest.

Previously, we and others have shown that loss of *Nf1 *gene function during murine embryogenesis causes defects of bone and cartilage development [9,10]. One of the observed molecular changes in *Nf1*-deficient embryonic cartilage was an upregulation and persistently nuclear localization of the transcription factor SOX9. Interestingly, SOX9 was also recently found to be expressed in NF1-related tumours, where it supports cellular survival [11]. As a master regulator of cartilage differentiation, SOX9 regulates expression of various downstream target genes, including collagen type 2a1, collagen type 11a2, aggrecan and melanoma-inhibitory activity (*MIA*). The last, *MIA*, is also known as cartilage-derived retinoic acid sensitive protein (*cd-rap*) and was originally isolated as a secretory factor from supernatants of melanoma cell cultures [12]. MIA serum level was found to correlate with melanoma spreading [13] and was proposed as a biomarker for monitoring the course of disease and the efficacy of therapies [14]. Various other tumours, predominantly those of neuroectodermal, glial origin, also express *MIA *[15]. Recombinant MIA inhibits melanoma cell growth and cell attachment *in vitro *[16]. Subsequent studies revealed that MIA interacts with extracellular matrix components, laminin and fibronectin, as well as with cellular matrix receptors integrin α5, integrin α4 [17] and cadherin 7 [18].

In the present study, we examined expression of *MIA *in *Nf1*-deficient mouse cartilage, in human cutaneous and plexiform neurofibromas and MPNSTs, and in sera of NF1 patients with these tumours. MIA in the serum of healthy probands was examined as a control.

## Methods

### Mouse breeding and tissue processing

The mice were continuously back-crossed to wild-type C57BL/6J to minimize the variation of genetic background. The female Nf1flox mice were crossed to male Nf1flox heterozygous Prx1-Cre-positive males and the offspring genotyped as previously described [9]. Embryos and postnatal tissue samples were fixed overnight at 4°C in 4% paraformaldehyde, dehydrated through an ethanol/xylol series, and embedded in paraffin blocks. Six-micrometer sections were cut and processed for haematoxylin and eosin/Alcian blue staining and *in situ *hybridization.

### Patients and samples

The study was conducted with a cohort of 42 NF1 patients and 22 healthy individuals. The diagnosis of NF1 was made using National Institutes of Health criteria. The study protocol was approved by the local institutional review board, and all patients gave their informed consent. Cutaneous and subcutaneous tumours were counted or estimated in case the number was larger than 100. Plexiform neurofibromas, including internal ones, were detected by means of whole-body MRI in 30 of the 42 patients. Because of the limited resolution of whole-body MRI, lesions smaller than 3 cm in the longest diameter, which is often the case for spinal tumours, were not included. Tumour sizes were calculated using a semiautomated volumetric method, and the total internal tumour load was obtained subsequently, including PNs (Plexiform Neurofibromas), spinal tumours and internal nodule neurofibromas, but excluding cutaneous and subcutaneous tumours [19]. An age effect for cutaneous, subcutaneous and internal tumours was examined using a nonparametric Spearman's rank-correlation test.

All serum samples were prepared using a standardized protocol in the laboratory of the Department of Maxillofacial Surgery at the University Medical Center Hamburg-Eppendorf. Whole blood of each patient was kept at room temperature for 30 minutes before being spun down at 4,500 rpm for 10 minutes using a benchtop centrifuge. The supernatant was stored at -80°C in 100-μL aliquots.

### *In situ *hybridization

*In situ *hybridization was performed on paraffin sections according to standard protocol [9]. Images were collected using a DMR HC microscope (Leica, Wetzlar, Germany) equipped with an AxioCam HRc camera (Zeiss, Jena, Germany) and evaluated using AxioVision 4.1 software (Zeiss, Jena, Germany).

### Immunohistochemical detection of MIA

Sections of six cutaneous and three plexiform neurofibromas, as well as seven MPNSTs, from a total of sixteen NF1 patients were stained with monoclonal anti-human MIA antibody (R&D Systems, McKinley Place NE, Minneapolis) diluted at 1:40. Sections were boiled in citrate buffer (pH 6.1) for antigen retrieval. The streptavidin-biotin method was performed using an automated staining system TechMate (Dako, Hamburg, Germany) with an implemented counterstaining. Negative controls were carried out with normal serum without the primary antibody or with antibody preincubated in access (25 ng/μl) of recombinant human MIA (Peprotech GmbH, Hamburg, Germany). Stained sections were analyzed using the BX51 microscope (Olimpus, Hamburg, Germany) and analySIS 5.0 software (Soft imaging system GmbH, Münster, Germany).

### Quantitative real-time polymerase chain reaction

RNA was isolated from the knee cartilage of two wild-type and two Nf1Prx1 mice using peqGOLD TriFast (PeqLab Biotechnologie GmbH, Erlangen, Germany) according to the supplied protocol. cDNA was synthesized from 1 μg of total RNA with MuLV Reverse Transcriptase (Applied Biosystems, Carlsbad, CA, USA). TaqMan Universal PCR Master Mix was then performed on an ABI PRISM 7900 Cycler (Applied Biosystems) using the SYBR Green method (Invitrogen, Darmstad, Germany) according to the manufacturer's instructions. The expression level of *Mia *was determined in Nf1Prx1 and wild-type tissues and was equilibrated against expression of glyceraldehyde 3-phosphate dehydrogenase (*GAPDH*). The following primers were used: mGAPDH: 5' GGGAAGCCCATCACCATCTT 3', 5' CGGCCTCACCCCATTTG 3'; mMIA: 5' GGAGGACCTGACTCTGAAACC 3'; 5' ACTGCAGGGATAGCGGTAG 3'.

### Mia elisa

The MIA ELISA kit was purchased from (Roche Diagnostics, Indianapolis, IN, USA) and the measurements were conducted in duplicate according to the supplied protocol. Internal negative and positive quality controls were provided in each enzyme-linked immunosorbent assay (ELISA) kit and were run in triplicate in each assay.

## Results

### *Mia *expression is elevated in *Nf1*-deficient murine cartilage

**Figure 1:**
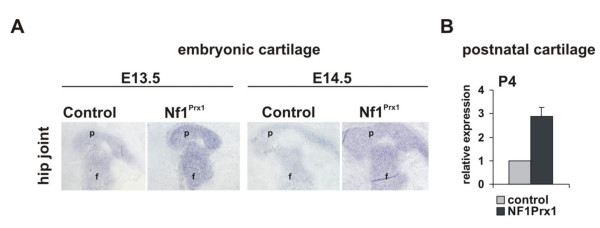
**Elevated expression of *cd-rap/Mia *in the *Nf1*-deficient cartilage**. **(A) ***In situ *hybridization of the melanoma-inhibitory activity/cd-rap (*mia*)-specific riboprobe on the transverse sections of E14.5 Nf1Prx1 embryos. Intensity of staining reflects abundance of *Mia *transcript. **(B) **Quantitative real-time polymerase chain reaction (qRT-PCR) of *Mia *transcript in the postnatal day 4 knee joints. Data represent means (± SD) of duplicate absolute quantifications for each probe. Transcript of the housekeeping gene glyceraldehyde 3-phosphate dehydrogenase (*GAPDH*) was used as control.

*In situ *hybridization revealed expression of *Mia *in the cartilage of the E14.5 to E15.5 mouse embryo (Figure 1). Expression domains of *Sox9, Col2a *and *Mia *overlapped and, in the E14.5 embryo sections, demarcated cartilage anlagen of the future bones (Figure 1A). The expression of *Mia *was found to be more intensive in the *NF1*-deficient cartilage of the Nf1Prx1 mice (Figure 1B). Similar results were obtained with mouse embryos bearing cartilage-specific inactivation of *Nf1 *(data not shown). We next quantified *Mia *expression by performing quantitative real-time polymerase chain reaction (qRT-PCR). Absolute quantification was conducted on the RNA isolated from knee cartilage of two mutant and two control mice at P4. *Mia *transcript levels in *Nf1*-deficient tissue were compared to the wild-type tissue and normalized to *GAPDH *expression. qRT-PCR revealed a more than twofold increase of *Mia *expression in Nf1Prx1-deficient cartilage.

### *MIA *is expressed in NF1-associated tumours

**Figure 2:**
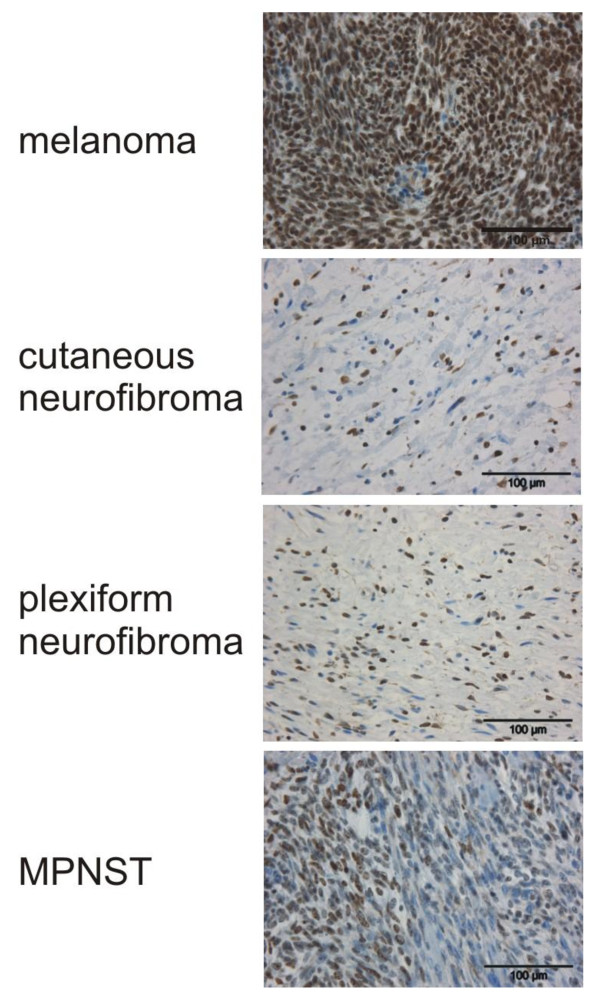
***MIA *is expressed in NF1 tumors**. Immunohistochemical detection of MIA on paraffin sections of NF1-associated tumors. Melanoma samples were used as positive controls. MIA is expressed in each type of the analysed NF1 tumors. Malignant peripheral nerve sheath tumours (MPNSTs) have higher cellular density, yielding more MIA-positive cells per visual field.

MIA was immunohistochemically detected on the paraffin sections of six cutaneous and three plexiform neurofibromas and in seven MPNSTs from NF1 patients. The typical pattern of MIA staining was a mixture of positive and negative nuclei side-by-side (Figure 2). The proportion of MIA-positive cells varied between 50% and 90%. The most intense staining was obtained in MPNSTs, which, however, represents the high density of nuclei in this type of tumour. No morphological difference was observed between MIA-positive and MIA-negative cells. On the basis of the degenerative nuclear atypia of Schwann cells, we deduced that MIA was both positive and negative in Schwann cell nuclei. MIA-positive cells were more often seen in areas of spindle-shaped cells arranged in fascicles.

### Serum concentration of MIA in NF1 patients correlates with tumour load

MIA serum level was determined in the 42 NF1 patients and in 22 healthy individuals. The patients' ages ranged from 14 to 72 years (mean age, 36 years). The control group's ages ranged between 19 and 67 years (mean age, 40 years). An age effect was seen in the NF1 patients for the number of cutaneous tumours (*P *= 0.023), but not for subcutaneous tumours (*P *= 0.842) or internal tumours (*P *= 0.449). Additionally, linear regression analysis revealed an association between total internal tumour load and the number of subcutaneous tumours (*P *value 8.19E-17 for the F test), but not between internal tumour load and the number of cutaneous tumours.

**Figure 3:**
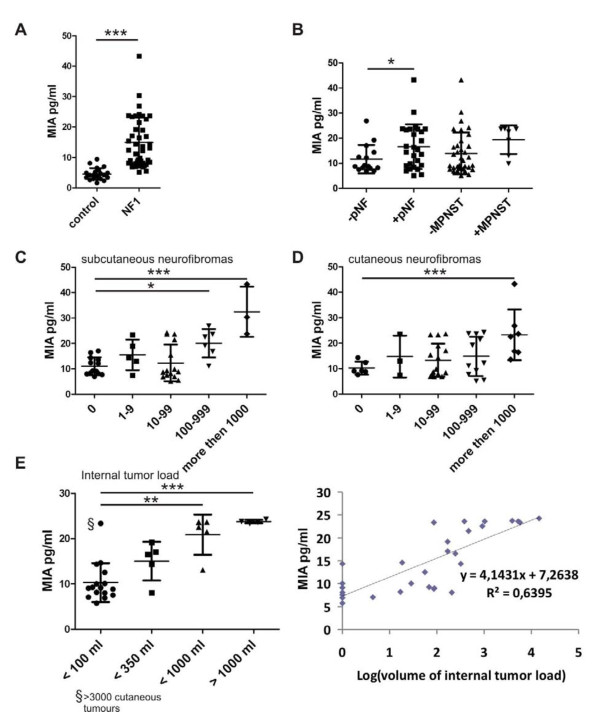
**MIA is elevated in serum from NF1 patients and reflects the internal tumor load**. **(A) **MIA serum levels in 42 NF1 patients and 22 healthy controls.** (B)** The 42 NF1 patients divided into subgroups according to the absence (-) or presence (+) of pNFs or MPNSTs. **(C, D) **The 42 NF1 patients were further divided with accordingt to cutaneous and subcutaneous tumors load. **(E) **In 30 of the 42 NF1 patients, internal tumor load was determined by whole-body magnetic resonance imaging (MRI). The 30 patients were arbitrarily divided into four groups according to the total tumor load: 0 to 100 mL (*n *= 16), < 350 mL (*n *= 5), < 1,000 mL (*n *= 5) and > 1,000 mL (*n *= 4). Differences between groups were evaluated using an unpaired *t*-test **(A and B) **or one-way analysis of variance (ANOVA) with a *post hoc t*-test including the Bonferroni correction **(C to E)**. **P < 0.01. ***P < 0.001. The linear regression analysis revealed a positive correlation between the logarithm of internal tumor load and MIA serum concentration.

MIA serum concentration was independent of age and sex (data not shown), but was significantly higher in NF1 patients than in healthy controls: 15.16 ± 1.26 pg/mL versus 4.54 ± 0.40 pg/mL (*P *< 0.001, unpaired *t*-test with Welch's correction) (Figure 3A). Among the 42 NF1 patients, the 27 patients with pNFs had significantly higher MIA serum concentration than the 15 patients without those tumours (*P *= 0.032) (Figure 3B). However, no significant difference in MIA serum level was found between the 7 and 35 patients with and without MPNSTs, respectively (Figure 3B). MIA serum level was also significantly higher in the nine and seven patients with > 100 subcutaneous neurofibromas and > 1,000 cutaneous neurofibromas, respectively, than in those without such tumours (Figures 3C and 3D). Internal tumour load was determined for 30 of the 42 NF1 patients on the basis of whole-body MRI. The patients were divided into four groups: very low internal tumour loads (0 to 100 mL; *n *= 16), low internal tumour loads (< 350 mL; *n *= 5), moderate internal tumour loads (< 1,000 mL; *n *= 5) and high internal tumour loads (> 1,000 mL; *n *= 4) (Figure 3E, left). One-way analysis of variance with the Bonferroni multiple comparison test revealed significant differences between MIA serum levels in patients with very low internal tumour loads and groups with high and very high internal tumour loads (**P < 0.01, ***P < 0.001). Also, linear regression analysis revealed an association between the total internal tumour load and MIA serum level (*P *value of 1.95E-7 for the F-test). The line that best predicts MIA level from values of logarithm of internal tumor load volume was identified by regression analysis: R^2^ = ~0.64 (Figure 3E, right). These data indicate that elevated MIA serum level may be indicative of an increased internal tumour burden. Since we observed an association between total internal tumour load and the number of subcutaneous tumours, a study involving a larger cohort size is necessary to reveal the relative contributions of internal, subcutaneous and possibly also cutaneous tumours to elevated MIA levels.

## Discussion

In this study, we found increased *Mia *expression in *Nf1*-deficient cartilage of Nf1Prx1 mice where SOX9 expression and nuclear localization were previously shown [9]. *MIA *promoter was previously shown to be regulated by SOX9 in a dose-dependent manner in cultured chondrocytes [20]. It thus appears likely that *MIA *expression in NF1 tumours is also regulated by SOX9, as this transcription factor was reported to be required for the survival of MPNST cells [11]. Our finding of MIA expression in various NF1-related tumours is consistent with the findings of previous reports that MIA is expressed in glial tumours [15].

The major finding of the present study is that MIA serum levels correlate with the internal tumour load in NF1 patients. Provided that this correlation can be confirmed in a larger cohort of NF1 patients, MIA would be a valuable biomarker for the internal tumour load.

In malignant melanoma cells, MIA was shown to bind integrin α5 and reduce ERK activity [17]. MIA/cadherin-7 interactions were shown to regulate cell-cell adhesion of malignant melanoma cells, influencing their migration [18]. It was also reported that MIA augmented transforming growth factor-β-mediated chondrogenic differentiation of human mesenchymal cells *in vitro *[21] and inhibited articular cartilage mineralization *in vivo *[22]. It will be interesting to examine whether any of these effects of MIA play a role in NF1-related tumorigenesis and skeletal dysplasia. While more studies are needed to understand the contribution of MIA to NF1 pathology, the presented correlation of MIA serum level with the internal tumour load suggests that it is a promising candidate as a biomarker of the tumour load in NF1.

## Conclusions

MIA is a potential biomarker of tumour load in NF1 patients and should be further evaluated for application in monitoring the clinical course and therapy outcomes of patients.

## Competing interests

The authors declare that they have no competing interests.

## Authors' contributions

MK formulated the hypothesis, coordinated the study, evaluated data and conceived the manuscript. VM provided clinical data and specimens. NK performed MIA ELISA measurements. RN performed whole-body MRI evaluations. JK provided expertise on the ELISA system handling and data acquisition. TZ performed statistical analysis. AJ helped in establishing the MIA immunohistochemistry protocol. EW performed real-time PCR experiments. BT performed *in situ *hybridization analysis. ST provided NF1 tumour samples and sera. NH provided logistical support and helped in collection of the serum samples. SP helped in collection of the serum samples. PB provided logistical support and helped in collection of the serum samples. DK provided support in obtaining serum probes and critically revised the manuscript. AH performed histological and immunohistological analysis of the surgically removed tumour material. SM critically revised the manuscript. LK coordinated clinical data and specimen acquisition, was involved in the evaluation and interpretation of data, and conceived and critically revised the manuscript. All authors read and approved the final manuscript.

## Pre-publication history

The pre-publication history for this paper can be accessed here:

http://www.biomedcentral.com/1741-7015/9/82/prepub
